# Textile industry and occupational cancer

**DOI:** 10.1186/s12995-016-0128-3

**Published:** 2016-08-15

**Authors:** Zorawar Singh, Pooja Chadha

**Affiliations:** 1 Department of Zoology, Khalsa College, G.T. Road, Amritsar, Punjab 143001 India; 2 Department of Zoology, Guru Nanak Dev University, Amritsar, Punjab India

**Keywords:** Textile industries, Cancer, Occupational cancer, Mutagenic, Mortality

## Abstract

**Background and summary:**

Thousands of workers are engaged in textile industry worldwide. Textile industry involves the use of different kinds of dyes which are known to possess carcinogenic properties. Solvents used in these industries are also associated with different health related hazards including cancer. In previous studies on textile and iron industries, the authors have reported genotoxicity among them and observed occurrence of cancer deaths among textile industry workers. Thus, an attempt has been made to compile the studies on the prevalence of different types of cancers among textile industry workers.

**Literature search:**

A wide literature search has been done for compiling the present paper. Papers on cancer occurrence among textile industry workers have been taken from 1976 to 2015. A variety of textile dyes and solvents, many of them being carcinogenic, are being used worldwide in the textile industry. The textile industry workers are therefore, in continuous exposure to these dyes, solvents, fibre dusts and various other toxic chemicals. The present study evaluates the potential of different chemicals and physical factors to be carcinogenic agents among occupationally exposed workers by going through various available reports and researches. Papers were collected using different databases and a number of studies report the association of textile industry and different types of cancer including lung, bladder, colorectal and breast cancer. After going through the available reports, it can be concluded that workers under varied job categories in textile industries are at a higher risk of developing cancer as various chemicals used in the textile industry are toxic and can act as potential health risk in inducing cancer among them. Assessing the cancer risk at different job levels in textile industries may be found useful in assessing the overall risk to the workers and formulating the future cancer preventive strategies.

## Background

Textile is one of the leading industries in the world. The textile industry workers are exposed to a number of chemicals including dyes, solvents, optical brighteners, finishing agents and numerous types of natural and synthetic fibre dusts which affect their health. Various dyes and solvents used by the textile industry have been found to have mutagenic and carcinogenic properties. Workers engaged in finishing processes are frequently exposed to crease-resistance agents. These agents may release formaldehyde which is known for its toxicity. Workers are also exposed to flame retardants including organophosphorus and organobromine compounds. The textile industries use different kinds of dyes including the most commonly used azo dyes which are aromatic hydrocarbon derivatives of benzene, toluene, naphthalene, phenol and aniline. The solvents used by the workers in different sections result in a major carcinogenic effect by direct contact with the subjects. A number of studies have been put forward emphasizing the occurrence of different types of cancers among textile industry workers [1–34]. Keeping in view the importance of the issue, a brief review of the same is presented herewith.

## Bladder cancer

Different studies have pointed out the occurrence of bladder cancer among textile industry workers [35–37]. Gonzales et al. [35] presented results from a case-control study carried out in the county of Mataro, Spain. The study was based on 57 cases that were hospitalized for or died from bladder cancer between 1978 and 1981. An increased risk for past employment in the textile industry (Odds ratio, OR = 2.2; *p* = 0.038) was found among a group of common occupational sectors. Further analyses in the study indicated that the risk for subjects who worked in dyeing or printing sectors and who were exposed to azo-dyes was particularly elevated (OR = 4.41; 95 % confidence limits; 1.15–16.84). Similarly, Zheng et al. [36] conducted a study on 1,219 incident bladder cancer cases based on gender which were diagnosed during the period 1980 to 1984. The bladder cancer cases were compared with 1982 census data on employment. Standardized incidence ratios (SIR) for bladder cancer were estimated for occupation and industry classifications and significant excess risks were observed for dyers, textile bleachers, and finishers (male: SIR = 169); metal refining and processing workers (male: SIR = 139; female: SIR = 197); apparel industry workers and workers engaged in other textile products manufacturing (female: SIR = 204). Serra et al. [37] also investigated the risk of bladder cancer in Spanish textile workers and analyzed the data from a multicenter hospital-based case-control study in Spain. The data included 1219 bladder cancer cases and 1271 controls. Out of those cases, 126 cases and 122 controls reported a history of previous employment in the textile industry. Increased risks were observed for weavers and workers engaged in winding, warping and sizing. Higher risk was also found for workers who were exposed to synthetic materials. Table 1 shows the incidence of different types of cancers among textile industry workers.

**Table 1:** Studies based on occurrence of different types of cancers among textile industry workers

Sr. No.	Study	Subjects	Type of cancer studied	Output of the study
1	Serra et al., 2008 [37]	Textile industry workers	Bladder cancer	Increased cancer risks were observed for weavers and for workers in winding, warping and sizing. Job more than 10 years appeared to be associated with an increased risk for weavers.
2	Li et al., 2015 [33]	Female textile workers	Breast cancer	No positive association between night shift work and breast cancer.
3	Li et al., 2013 [48]	Female textile workers	Breast cancer	No association was observed between cumulative exposure to MFs and overall risk of breast cancer.
4	Ray et al., 2007 [25]	Female textile workers	Breast cancer	Endotoxin or other components of cotton dust exposures may be associated with reduced risks for breast cancer
5	Fang et al., 2013 [46]	Textile workers	Cancer mortality	Mortality risk from gastrointestinal cancers and all cancers combined, with the exclusion of lung cancer, were increased in cotton workers as compared to silk workers.
6	Wang et al., 2012 [42]	Asbestos textile workers	Cancer mortality	Highest cancer mortality was observed in the high exposure group, with 1.5-fold age-adjusted mortality from all cancers and 2-fold from lung cancer compared to the low exposure group.
7	Kuzmickiene and Stukonis, 2010 [49]	Female flax textile workers	Oral cavity and pharynx cancer	Risk of oral cavity and pharynx cancer was significantly increased in spinning-weaving unit workers with <10 years of employment (SIR 5.71, 95 % CI 1.56 to 14.60).
8	Gunay and Beser, 2011 [50]	Turkish textile workers	Early breast cancer	91.6 % of the women working in a textile factory in Turkey had no education about breast cancer.
9	Kwon et al., 2015 [32]	Female textile workers	Lung cancer	No increased risk of lung cancer among rotating shift workers.
10	Checkoway et al., 2015 [51]	Female textile workers	Lung cancer	Reply to [34]: Exposure–response association may change over time owing to complex, yet poorly understood, underlying mechanisms. Endotoxin is a highly variable exposure, and as we noted in the paper, some exposure misclassification was inevitable.
11	Rylander and Jacobs, 2015 [34]	Female textile workers	Lung cancer	In comment to [30]: The result should be “no relation between endotoxin exposure and lung cancer risk could be detected”
12	Checkoway et al., 2014 [30]	Female textile workers	Lung cancer	The study did not support a protective effect of endotoxin, but is suggestive of possible lung cancer promotion with increasing time since first exposure.
13	Wang et al., 2014 [31]	Textile and mining workers	Lung cancer	A clear exposure-response relationship between lung cancer mortality and exposure levels.
14	Applebaum et al., 2013 [44]	Female textile workers	Lung cancer	A reduced cancer risk in workers exposed to endotoxin, hired >35 years before enrolment [IRR = 0.74, 95 % CI (0.51 to 1.07)] as compared to hired </=35 years.
15	Gallagher et al., 2013 [52]	Female textile workers	Lung cancer	Cancer risk was higher in women with a surgical menopause (HR = 1.64, 95 % CI 0.96–2.79) than in those with a natural menopause (HR = 1.35, 95 % CI 0.84–2.18) demonstrating biological role of hormones in lung carcinogenesis.
16	Agalliu et al., 2011 [39]	Female textile workers	Lung cancer	Endotoxin exposure that occurred 20 years or more before risk confers the strongest protection against lung cancer, indicating a possible early anti-carcinogenic effect.
17	Checkoway et al., 2011 [38]	Female textile workers	Lung Cancer	No associations were observed for lung cancer with wool, silk or synthetic fibre dusts. Increased risks were noted for >/= 10 year exposures to silica (adjusted HR 3.5, 95 % CI 1.0 to 13) and >/= 10 year exposures to formaldehyde (adjusted HR 2.1, 95 % CI 0.4 to 11).
18	Astrakianakis et al., 2010 [53]	Female textile workers	Lung Cancer	A dose-related inverse lung cancer risk was associated with cumulative endotoxin exposure but a possible anti-carcinogenic effect at early stages of lung cancer pathogenesis was not evident.
19	Lenters et al., 2010 [29]	Agriculture industry and cotton textile workers	Lung Cancer	Occupational exposure to endotoxin in cotton textile production and agriculture is protective against lung cancer
20	Loomis et al., 2009 [28]	Asbestos textile workers	Lung Cancer	Mortality from all causes, all cancers and lung cancer was significant higher than expected, with SMRs of 1.47 for all causes, 1.41 for all cancer and 1.96 (95 % CI 1.73 to 2.20) for lung cancer.
21	Kuzmickiene and Stukonis, 2007 [24]	Textile workers	Lung Cancer	Exposure to cotton textile dust at workplaces for male is associated with adverse lung cancer risk effects but lung cancer risk decreased with level of exposure to textile dust.
22	Loomis et al., 2012 [54]	Asbestos textile workers	Lung Cancer	Lung cancer is associated most strongly with exposure to long thin asbestos fibres. Fibres 5–10 μm long and <0.25 μm in diameter were associated most strongly with lung cancer mortality.
23	Elliott et al., 2012 [41]	Asbestos textile workers	Lung Cancer	Increased rates of lung cancer were significantly found to be associated with overall cumulative fibre exposure.
24	Wernli et al., 2008a [27]	Textile workers	Endometrial cancer	An increased risk of endometrial cancer was detected among women who had worked for > or =10 years in silk production (HR = 3.8, 95 % CI 1.2–11.8).
25	Wernli et al., 2008b [55]	Textile workers	Ovarian cancer	An increasing risk of ovarian cancer associated with cumulative exposure to silica dust (for <10 years exposure, HR = 6.8 [CI = 0.6–76]; for > or =10 years, 5.6 [1.3–23.6]).

*SIR* standardized incidence ratios, *MFs* magnetic fields, *HR* hazard ratio, *IRR* incident rate ratios, *SMRs* standardized mortality ratios

## Lung cancer

A number of studies report the association of textile industry and lung cancer. The association between endotoxin exposure and lung cancer risk was found in a cohort of female textile workers [23]. Bacterial endotoxin which is a contaminant of raw cotton fibre and cotton dust, has been proposed as a protective agent against cancer. The action of endotoxin may be through the innate and acquired immune systems. Long-term and high-level exposure to endotoxin, compared with no exposure was found to be associated with a reduced risk of lung cancer in this cohort. Similarly, Checkoway et al. [38] investigated the associations of various exposures like wool, synthetic fibre dusts, formaldehyde, silica, dyes and metals with lung cancer in the textile industry. But in this study, no associations were observed for lung cancer with wool, silk, synthetic fibre dust or with other agents. Agalliu et al. [39] investigated the associations between contiguous windows of endotoxin exposure and risk of lung cancer, and reported that endotoxin is consistently associated with a reduced risk of lung cancer. Data from 602 cases of female textile workers was evaluated in Shanghai, China and an inverse risk trend of lung cancer with increasing levels of endotoxin exposure was found. In a study of Italian textile workers (*N* = 1966), on the basis of 68 deaths from mesothelioma, the standardized mortality ratio (SMR) was found to be 6627 for workers employed only under the age of 30 years. SMR was found to be 8019 for workers those were employed both under the age of 30 years and at the age of 30–39 years. SMR was 5891 for those employed both under the age of 30 years and at the age of 40 years or more. The results of the study also indicated that stopping the exposure of the workers does not modify the subsequent mesotheliomas risk [40].

Elliott et al. [41] conducted a study in North and South Carolina on two US cohorts of asbestos textile workers exposed to chrysotile. The study found an increasing risk of lung cancer mortality with cumulative fibre exposure. Similarly, Wang et al. [42] determined the mortality associated with exposure to chrysotile asbestos from a textile factory in China. The study was done from 1972 to 2008 and a total 577 workers were followed. Follow-up rate for the study was 98.5 % over 37 years. The follow-up of the workers generated a data of 17,508 persons including 259 deaths (from all causes), 2 mesotheliomas and 53 lung cancers. The highest cancer mortality was observed in the high exposure group, with 1.5-fold age-adjusted mortality from all cancers and 2-fold from lung cancer when compared to the low exposure group. Both smokers and non-smokers at the high exposure level had a high death risk from lung cancer. A clear exposure-response trend was seen in smokers which confirmed an increased mortality from lung cancer and all cancers in asbestos workers and the cancer mortality was found to be associated with exposure levels. Deng et al. [43] described mortality in workers exposed to chrysotile asbestos and determined exposure-response relationships between asbestos exposure and mortality from lung cancer. A cohort of 586 workers in an asbestos textile factory was followed. Individual cumulative asbestos exposure was estimated as the product of fibre concentrations and duration of employment in each job and expressed as fibre-years/ml (e.g., 30 fibre-years/ml is an exposure equivalent to 30 years of exposure at 1 fibre/ml concentration or 15 years at 2 fibres/ml; and so on). It was found that out of the 226 deaths, 51 deaths were from lung cancer and 37 from asbestosis. A significant exposure-response relationship between asbestosis and lung cancer (*p* < 0.001) was observed. Applebaum et al. [44] also examined the relationship between endotoxin and lung cancer in a study of Chinese female textile workers. Enrollment of the workers was done between 1989 and 1991 and the workers were followed till 1998. In the study, 3038 sub-cohort members and 602 incident lung cancer cases were analyzed. Among the workers, who were never exposed to endotoxin, a comparison was made between lung cancer rates in workers hired more than 35 years before enrolment and workers hired less than or 35 years before enrolment. In the former group, a reduced risk (Incidence rate ratio, IRR = 0.74, 95 % CI) was found. An increased risk of lung cancer among workers hired for more than 50 years ago was also reported.

Dement and Brown [12] investigated the causes of deaths among textile workers and found 185 excess deaths (SMR = 1.44) out of a mortality of 1200 South Carolina textile workers. These excess deaths included 41 lung cancers (SMR = 2.25), 43 non-malignant respiratory diseases (SMR = 2.25) and 71 cardiovascular diseases (SMR = 1.37). In whole of the study, only two mesotheliomas cases were observed. Simpson et al. [45] examined the relation between women’s health and their occupation. The study analyzed the data of 381,915 women cancer cases which were registered in England from 1971 to 1990, over the period of 20-year. For exploring the value of the data, five sites (lung, pleura, bladder, breast and stomach) under two occupations including agriculture and textile were selected. The association between stomach cancer and “dusty” occupations were found to as PRR (Proportional registration ratios) = 198, 95 % (CI = 126–298) for textile finishers. Similarly, Mastrangelo et al. [14] analyzed textile industry workers to evaluate the cancer risk within the textile industry in relation to the textile fibre being used or the specific type of job held in the industry. The decrease in the cases of upper respiratory tract cancer paralleled with a corresponding increase in the cases of lung cancer. Conclusively, the importance of preventive measures to reduce the lung cancer burden in the textile workers was emphasized.

## Other cancer types

Apart from occurrence of bladder and lung cancer cases in textile industry workers, various other cancer types are also reported in different studies. Camp et al. [15] assessed the development of a cancer study among Shanghai textile workers. The results of the study indicated that women employed in wool, cotton, mixed-fiber and machine-maintenance sectors have a significantly increased risk for breast cancer. De Roos et al. [17] investigated the probable risks of rectum and colon cancers in relation to different types of exposures in textile industry. The investigation revealed that certain long term exposures in textile industry may pose an increased risk of colorectal cancers. Hazard ratio for exposures especially to textile dyes and their intermediates with colon cancer was found to be HR = 3.9; 95 % CI: 1.4–10.6 (> or =20 years exposure versus never). In the same way, Chang et al. [19] investigated the associations between biliary tract cancer (BTC) and occupational exposures to various chemicals and textile dusts in a cohort of 267,400 women textile workers. For employment in maintenance jobs, an increased risk of BTC was found (HR = 2.92, 95 % CI: 1.48, 5.73) with a significant trend by duration of exposure. It was also suggested that long-term exposures to different metals and employment in maintenance sector in the textile industry may have played a role in elevating the BTC risks among textile industry workers. Fang et al. [46] investigated the cancer mortality in relation to cotton dust and endotoxin exposure in a cohort from Shanghai textile workers by assessing 444 cotton textile workers. A reference group of 467 persons who were unexposed silk workers was also recruited. Both the groups were followed for 30 years. Hazard ratios for all cancers (with and without lung cancer) and gastrointestinal cancer were estimated in Cox regression models. In comparison to silk workers, cotton workers were found to have increased risks of mortality from gastrointestinal cancers and all cancers combined [gastrointestinal cancer HR = 4.1 (1.8–9.7); all cancers HR = 2.7 (95 % CI 1.4–5.2)]. A previous study by the present author also demonstrated genotoxic risk among textile industry workers [47].

## Conclusion

Textile industry workers are exposed to a number of chemicals which are known to have carcinogenic properties. Reviewing the data of 54 research papers on textile industry workers revealed the occurrence of different types of cancers among them. Exposure to different sets of chemicals and physical factors in textile industry may induce occupational cancer as a long term effect among textile industry workers. Formulation and use of alternate non-toxic textile chemicals for different processes should be encouraged. Conclusively, proper protection equipments and other precautionary measures should be used by the workers while dealing with toxic chemicals in these industries.

## Abbreviations

BTC: Biliary Tract Cancer
HR: Hazard Ratio
IRR: Incident Rate Ratios
MFs: Magnetic Fields
OR: Odds Ratio
PRR: Proportional Registration Ratios
SIR: Standardized Incidence Ratios
SMRs: Standardized Mortality Ratios
